# Supplementary material for: Are carbon emissions trading and green financial instruments synergistic? -Comprehensive quantitative research based on content analysis
Source: PLoS One. 2024 Mar 7;19(3):e0298601. doi: 10.1371/journal.pone.0298601 (PMC10919601; doi:10.1371/journal.pone.0298601)
Supplement: S1 Data — (ZIP) [file pone.0298601.s001.zip › Data/地级市Z_F工作报告.zip!/╔╜╢1⁄2/╟α╡║╩╨/└┤╫╘╣1⁄2╓┌║┼í╛┬φ┐╦╩2╛▌═°í┐_2018.docx]

各位代表：

　　现在，我代表市人民政府向大会报告工作，请予审议，并请各位政协委员和其他列席人员提出意见。

　　一、2017年工作回顾

　　2017年是全市上下凝心聚力、拼搏奋进的一年。我们坚持以习近平新时代中国特色社会主义思想为指导，在省委省政府和市委的坚强领导下，深入学习贯彻党的十八大、十八届历次全会和十九大精神，按照市第十二次党代会和市第十六届人大一次会议确定的目标任务，坚持稳中求进工作总基调，以供给侧结构性改革为主线，开拓进取，扎实工作，经济社会实现持续健康发展。

　　预计，全市生产总值实现11000亿元左右，同比增长7.5%左右；一般公共预算收入达到1157.1亿元，增长7.1%；固定资产投资增长7.5%左右；社会消费品零售总额增长10.5%左右；全体居民人均可支配收入增长8%左右；城镇登记失业率3.1%；居民消费价格上涨2%左右；完成节能减排降碳任务。主要做了以下工作：

　　（一）坚持综合施策、精准发力,经济增长呈现新态势。面对下行压力，强化各级抓发展责任，加强研判调度和政策引导，帮助企业解决问题，实现经济发展稳中有进。200个市级重点建设项目全部开工，完成投资1726亿元。1200个亿元以上产业项目加快推进。实施“十大消费促进工程”，对经济增长的基础作用进一步增强。规上工业增加值增长7.5%左右。服务业增加值增长8%左右，占生产总值比重达55%以上。粮食产量296.9万吨，肉蛋奶产量100万吨，蔬菜产量830万吨，水产品产量121万吨。落实“三去一降一补”任务，金融风险防范、化解和整治取得实效。推进“营改增”改革，落实小微企业税收优惠，清理规范涉企收费和基金，全年为企业减少地方税费180亿元。

　　（二）坚持提质增效、转型升级，动能转换实现新突破。积极培育新动能，推进1000个重大项目，一批事项纳入省新旧动能转换综合试验区建设总体方案。西海岸新区、高新区、蓝谷等重点功能区加快崛起。保税港区整合优化方案获国务院批复。出台培育市场主体发展民营经济的政策措施，新登记市场主体25.1万户，总量达到118.7万户。实施“双百千”行动和“一业一策”计划，出台现代服务业、高技术产业、工业、农业、建筑业五大领域98个行业行动计划。滚动推进总投资2488亿元的432个工业转型升级项目，一汽大众华东生产基地、上汽通用五菱乘用车等110个项目竣工。战略性新兴产业和高技术产业增加值分别增长10%、11%左右。海尔COSMOPlat等17个平台和项目入选全国制造业与互联网融合发展等试点示范专项。成为“中国制造2025”试点示范城市。金融业增加值增长8%左右，旅游消费增长15%左右。国家现代农业示范区建设水平在副省级以上城市中居第二位。

　　（三）坚持创新驱动、人才支撑，核心竞争力有了新提高。国家高速列车技术创新中心、海洋国家实验室、国家虚拟现实产业基地、国家海洋基因库等平台加快建设。一批企业在技术、模式、业态创新方面成果丰硕，由海尔牵头制定的大规模定制标准成为全球首个由中国企业主导的智能制造国际标准，海信智能交通、光模块技术走在国内外同行前列。新引进4所大学在青设立分支机构。成为国家科技成果转移转化示范区、国家知识产权强市创建市。高新区获批国家“双创”示范基地。新增国家级孵化器、众创空间33家。国际人力资源服务产业园建成启用。高新技术企业2039家，增长51%。万人有效发明专利拥有量23.75件。海水稻实现测产。新引进各类人才13.1万人，新增住青院士5人、聘任院士3人、泰山系列人才工程专家66人，人才总量180万人。

　　（四）坚持深化改革、扩大开放，发展活力得到新增强。争取国家和省试点16项。深化“放管服”改革，市级行政权力事项精简56.5%，审批事项精简58.9%。推进“多证合一”，设立企业更加便利。市直企业资产、收入、利润总额分别增长22.7%、23.6%、24.2%。财富管理金融综合改革试验区建设不断深入，新引进金融机构13家，全球金融中心排名继续位居前50名。完成3941个村集体产权制度改革，农村产权交易额11.6亿元。平度国家中小城市综合改革试点成效显著。

　　实施国际城市战略取得新进展。积极开展“千企招商大走访”，市区（市）两级共走访企业8280家，新增签约项目642个。实际利用外资77.3亿美元，增长10%左右，总量占全省45%左右。实际到位内资1887.2亿元，增长13.3%左右。国际贸易总额达到1150亿美元，增长14%左右，其中货物进出口总额5007亿元，增长15%左右；服务进出口总额140亿美元，增长16%左右。位列全国服务外包示范城市前10强。新开通4条空中洲际直达航线。港口货物和集装箱吞吐量分别达到5.1亿吨、1830万标箱，青岛港全自动化码头投入使用。深度融入“一带一路”建设，对沿线国家投资额、进出口额分别占全市的48.3%、49%左右。新增1个国际友好合作关系城市、17个国际经济合作伙伴城市。建成7个境外青岛工商中心。成功举办亚欧数字互联互通高级别论坛、亚洲媒体峰会、金砖国家协调人第二次会议、国际教育信息化大会、国际标准化论坛等重要国际活动。

　　（五）坚持完善功能、强化治理，城市品质实现新提升。制定实施建设美丽青岛三年行动计划。中心城区实现控制性详规全覆盖。成为全国首批城市设计试点城市。完成即墨撤市设区区划调整。胶东国际机场建设进展顺利，配套交通体系建设全面启动。济青高铁、青连铁路、董家口疏港铁路加快建设。6条地铁线在建，2号线东段开通试运营，11号线空载试运行。打通16条未贯通道路。开展交通秩序大整治，实施公共交通换乘优惠，开辟调整22条市区公交线路，具备安全通行条件的行政村全部通公交车（客车）。新增公共停车泊位10045个。棘洪滩水库至董家口引水工程、山洲水库调水工程、胶河调水二期工程相继竣工。完成120个小城镇（中心村）、28个贫困村电网改造升级。海绵城市、地下综合管廊国家试点成效明显。成为全国装配式建筑示范城市。拆除违法建设880万平方米。创建星级农贸市场40处。新建改建主城区公厕157座，改造农村无害化厕所51.5万座。创建国家特色小镇2个、全国美丽乡村和中国最美休闲乡村11个。常住人口城镇化率71.53%。

　　以中央环保督察、国家海洋督察为契机，着力解决各类环境问题1638个。PM2.5、PM10平均浓度分别改善17.8%、10.6%。河长制、湾长制全面推行，胶州湾海域优良水质面积达到71.8%，完成14处黑臭水体治理。新建改建4个污水处理厂投入运行。农业面源污染治理取得新成效，禁养区内1095处养殖场全部关闭搬迁。新增造林3万亩，新建改建绿地458公顷，其中主城区366公顷。节能保暖改造339万平方米。新建天然气管网217公里。新增清洁能源供热1300万平方米。购置投运914辆新能源公交车。

　　（六）坚持加大投入、夯实基础，群众生活得到新改善。全市财政用于民生支出达到1058亿元，占总支出的75.4%。市办实事全部完成。城镇新增就业73万人，政策性扶持创业2.05万人。企业退休人员养老金实现“十三连涨”。城乡低保标准进一步提高，共为7.4万户低保对象发放救助资金7.4亿元。完成住房保障7052套（户），实施棚户区改造50677套（户），完成农村危房改造1050户。为14732户已入住居民补办不动产权证。完成636个村庄饮水安全巩固提升工程。全市149个省定贫困村、231个市定经济薄弱村摘帽。对口支援和扶贫协作工作扎实推进。完成100所标准化幼儿园建设、100所农村义务教育薄弱学校改造，新建82所中小学标准化食堂。5所寄宿制普通高中投入使用。八医东院区开工建设，市立医院东院二期、青大附院东院病房楼主体完工，新建16个院前急救站。公立医院全面取消药品加成，为群众节省药费21亿元。生育政策稳妥落实。养老体系建设不断加强，新增专业化养老服务机构11家，建成10处失智老人照护示范中心。市社会福利院、青岛优抚医院、市儿童福利院改扩建工程和市老年活动中心主体完工。成为国家食品安全示范城市。安全生产形势总体平稳。城市应急体系更加完善。信访积案和矛盾纠纷加快化解。推进公安基层基础建设，“一村一警务助理”实现全覆盖，反恐维稳措施不断强化，社会保持安全稳定。

　　文化青岛建设成效显著。我市蝉联全国文明城市，胶州成为全国县级文明城市。获全国“五个一”工程奖1项。电影金凤凰奖颁奖典礼、电影交易博览会成功举办，被联合国教科文组织授予“电影之都”称号。举办市民文化艺术节，6万多项文化活动惠及基层群众。获评全国十大数字阅读城市。我市运动员在第十三届全运会取得历史最好成绩。成功举办2017青岛马拉松等赛事。荣获世界帆船运动发展突出贡献奖。双拥共建深入开展。统计、档案、史志工作取得新成效。司法行政、仲裁作用进一步发挥。侨务、对台和民族宗教工作得到加强。人防、气象、防震减灾工作有序开展。妇女儿童、青少年、老龄、残疾人、红十字、慈善事业健康发展。

　　（七）坚持依法行政、转变作风，政府自身建设取得新进展。把学习贯彻党的十九大精神作为首要政治任务，着力在学懂弄通做实上下功夫。切实增强“四个意识”，严守政治纪律和政治规矩。“两学一做”学习教育常态化制度化扎实推进。认真落实向人大报告、向政协通报制度。坚决执行人大及其常委会的决议、决定，自觉接受人大法律监督、工作监督和政协民主监督。推动出台山东省青岛西海岸新区条例。向市人大常委会提交地方性法规草案5件，制定政府规章9件、修改27件、废止84件。修订完善市政府工作规则。行政执法体制改革取得显著成效。完成相对集中行政复议权改革。公共法律服务体系覆盖全市。政务公开工作全面推进。公布1791项市级公共服务事项目录和服务指南，取消、调整各类证明、盖章类材料70项。4519项政务服务事项实现“最多跑一次”，其中1878项实现“零跑腿”。市政府领导班子成员联系代表委员、联系企业、联系重点项目和走访企业活动深入开展。认真开展“向市民报告、听市民意见、请市民评议”活动，网络在线问政、行风在线、政务服务热线等政民互动渠道更加畅通。严格落实中央八项规定精神和实施细则，坚决纠正“四风”。落实“一岗双责”，推进政府系统廉政建设和反腐败斗争，着力整治侵害群众利益的不正之风和腐败问题，严肃查处了一批违纪违法案件。

　　各位代表！过去一年取得的成绩，是在习近平新时代中国特色社会主义思想指导下、省委省政府和市委坚强领导的结果，是市人大、市政协和社会各界监督支持的结果，是全市人民团结奋斗的结果。在此，我代表市人民政府，向全市人民，向全体人大代表和政协委员，向各民主党派、工商联、各人民团体、社会各界人士和离退休老同志，向中央、省驻青单位，向驻青部队和武警官兵，向所有关心支持青岛发展的海内外朋友们，表示衷心的感谢和崇高的敬意！

　　在肯定成绩的同时，我们也清醒地看到，发展不平衡不充分的问题尚未得到有效解决，经济发展的质量效益有待提升，发展环境仍需优化。实体经济发展还存在一些困难，产业大项目不足，民间投资、制造业投资不旺。创新主体的动力和能力还不够强，动能接续转换还不到位。区域之间、城乡之间发展不够协调。安全生产、生态环保以及社会事业等方面都还存在一些短板。文明城市建设还有待进一步深化。政府工作距离群众要求还有差距，一些公务人员的能力本领、担当精神和实干作风有待进一步加强，一些领域的消极腐败现象时有发生。我们一定正视问题、直面挑战，坚决采取措施积极应对、积极解决。

　　二、2018年目标任务

　　各位代表！中国特色社会主义进入新时代，要求我们必须有新气象新作为。我们要适应新时代、聚焦新目标、落实新部署，抢抓青岛发展新机遇，积极应对新挑战，以永不懈怠的精神状态和一往无前的奋斗姿态，谱写新时代中国特色社会主义的青岛篇章。

　　今年是贯彻党的十九大精神的开局之年，是改革开放40周年，是决胜全面建成小康社会、实施“十三五”规划承上启下的关键一年。政府工作总的要求是：全面贯彻党的十九大精神，以习近平新时代中国特色社会主义思想为指导，深入贯彻落实习近平总书记视察山东重要讲话、重要指示批示精神，坚持稳中求进工作总基调，坚持新发展理念，按照高质量发展的要求，统筹推进“五位一体”总体布局和协调推进“四个全面”战略布局，以供给侧结构性改革为主线，突出抓重点、补短板、强弱项，落实好“创新+三个更加”目标要求和“一三三五”工作举措，统筹推进稳增长、促改革、调结构、惠民生、防风险各项工作，推动质量变革、效率变革、动力变革，促进经济平稳健康发展和民生福祉持续改善，努力在实现社会主义现代化新征程中率先走在前列。

　　主要预期目标是：全市生产总值增长7.5%左右，一般公共预算收入增长6.5%左右，固定资产投资增长8%左右，社会消费品零售总额增长10%左右，货物进出口增长6%左右，全体居民人均可支配收入增长8%左右，城镇登记失业率控制在4%以内，居民消费价格涨幅控制在3%左右，完成年度节能减排降碳任务。

　　工作中，我们将把握好以下方面：

　　——牢牢把握率先走在前列的目标定位。强化走在前列的责任担当，坚持跳出青岛看青岛、站在未来看现在，提升思想境界、提升工作标准、提升责任担当，通过坚持不懈、持之以恒的努力，当好全省经济发展的龙头，积极创造条件争创国家中心城市，建设国际海洋名城。

　　——牢牢把握发展第一要务。把发展作为解决一切问题的基础和关键，坚定不移贯彻新发展理念，把推动高质量发展作为根本要求，使创新成为第一动力、协调成为内生特点、绿色成为普遍形态、开放成为必由之路、共享成为根本目的，努力实现更高质量、更有效率、更加公平、更可持续的发展。

　　——牢牢把握创新第一动力。把创新作为青岛今后发展的根本出路所在，紧紧抓住创新这个“牛鼻子”，形成引领城市发展的强大引擎，奋力把青岛建设得更加富有活力、更加时尚美丽、更加独具魅力。

　　——牢牢把握新旧动能转换的任务要求。深化供给侧结构性改革，以新技术、新产品、新产业、新业态为重点，大力培育新动能，改造提升传统动能，增强城市竞争力，当好全省实施新旧动能转换重大工程的排头兵、驱动器、示范区。

　　——牢牢把握以人民为中心的发展思想。始终把人民利益摆在至高无上的地位，以造福人民为最大政绩，把人民群众的小事当作自己的大事，从人民群众关心的事情做起，从让人民群众满意的事情做起，让改革发展成果更多更公平惠及全市人民。

　　三、致力于增强创新第一动力推动高质量发展，加快现代化经济体系建设

　　坚持质量第一、效益优先，深化供给侧结构性改革，推进新旧动能转换重大工程，促进经济发展质量效益和规模总量快速提升。

　　（一）抓好现代化产业体系建设。深入实施“双百千”行动和“一业一策”计划，支持本地企业发展壮大，吸引市外企业来青发展，争取更多的企业、行业主营业务收入进入百亿元和千亿元行列，加快形成实体经济、科技创新、现代金融、人力资源协同发展的产业体系，建设具有国际竞争力的先进制造业基地、国家重要的区域服务业中心和国际海洋名城。

　　做大做强战略性新兴产业和高技术产业。加快培育数字经济、生物经济、现代供应链等新业态新模式。落实国家大数据战略，推动互联网、大数据、物联网、人工智能和实体经济深度融合，力争大数据、云计算产业核心产值增长40%以上。统筹创新链、产业链、资金链和政策链，培育壮大集成电路、轨道交通装备、新能源汽车、海洋工程装备、航空航天等新兴行业。瞄准生物医药、医疗器械及仪器仪表、信息服务等行业，打造高技术产业集群。

　　做精做活现代服务业。加快服务业创新发展，建设服务业中心城市，培育行业领军企业，推动生产性服务业向专业化高端化提升、生活性服务业向智慧化品质化转变、公共服务业向规范化便利化发展。营造良好的金融生态，把握好服务实体经济、防控系统性风险、扩大对外开放等重点，推动驻青金融机构又好又快发展，在充分体现财富管理特色的同时，推动我市金融各行业持续快速健康发展。积极培育冷链物流、电商物流、供应链物流等物流新业态。建设港航贸易、交易、金融以及其他功能性平台，打造我国北方重要的国际航运中心。加快既有优势旅游项目建设，加强旅游大项目引进，打造满足多样化需求的精品旅游线路，搭建旅游大数据平台，面向国内外精准营销，办好世界旅游城市联合会青岛峰会，打造一流滨海度假旅游胜地。加强内部挖潜和外部引进，尽快做大会展业规模。加快培育健康服务业，创建国家健康产业发展试验区。积极发展法律、会计审计、检验检测、人力资源等商务服务业，完善与国际接轨的高端服务体系。

　　做优做新传统产业。高度重视传统产业对发展经济和改善民生的重要作用，高度重视传统产业与时俱进转型升级。加快批发、零售、住宿、餐饮、交通运输、仓储、邮政快递以及其他居民服务业发展，在供应内容、方式和便利性上更好地适应消费需求，争创国际消费中心城市。推进空客直升机等总投资2000亿元的300个工业转型升级项目，加快新一轮技术改造和产品升级。推进互联网工业“555”项目建设，促进制造业与互联网融合发展。实施中小企业创新转型行动，培育一批中小企业“隐形冠军”和制造业单项冠军。争创“中国制造2025”国家级示范区。

　　大力发展海洋经济。立足海洋优势和特色，积极融入和服务海洋强国战略，加快海洋高端创新载体和人才集聚，强化产业支撑能力，建设海洋科技创新强劲、海洋经济发达、海洋文化浓郁、海洋生态环境优美、对外开放程度高的国际海洋名城。提升海洋国家实验室、深海基地、深海钻探基地、海洋设备质检中心等国字号平台的创新引领和辐射服务能力，支持中国科学院海洋大科学研究中心建设，促进海洋科研机构协同创新，打造有影响力的海洋科技创新中心。发展海洋渔业，建设现代化海洋牧场，打造蓝色粮仓。支持海洋矿业、油气业、化工业、电力业和海水利用业发展。优化结构、创新方式、强化招商，加快发展海洋生物医药、船舶制造业和工程建筑业，加快发展海洋交通运输业、滨海旅游业。加快发展海洋相关产业。推进海洋特色产业园建设，着力培育海洋经济领军企业，打造海洋科技产业集聚示范区。壮大海洋高等教育规模和实力，提升海洋科学研究能力和水平，打造高水平海洋科研教育服务基地。统筹海洋深度开发和海岸高效利用，加快深远海开发战略保障基地建设，拓展蓝色发展空间。

　　大力发展实体经济。实体经济始终是我市经济发展的根基，必须高度重视并不断提高质量、规模、效益和竞争力。加强对固定资产投资的监测与调度，抓好200个市级重点建设项目，完成投资1600亿元以上，实现产业投资特别是制造业投资稳定增长。落实“三去一降一补”重点任务，在“破、立、降”上下功夫，不断提高供给质量。降低制度性交易成本，落实好国家税费改革政策，积极争取要素成本降低的任何可能机会，努力降低企业成本。加强金企对接和政策引导，鼓励金融机构大力支持实体经济特别是中小微企业。积极推动企业债转股，鼓励企业多渠道直接融资。

　　（二）抓好各类市场主体培育。激发各类市场主体活力，推进“个转企、小升规、规改股、股上市”，在培育铺天盖地的中小微企业的同时，努力培育更多顶天立地的大型企业和开天辟地的创新型企业。

　　扩大市场主体特别是企业数量。加强政策引导和工作力度，推动市场主体总量持续提升。市场主体总量年底突破130万户，其中企业超过47万户。支持注册新企业，完善“个转企”政策和工作机制。推动外地企业驻青分公司和办事机构转变为法人企业。

　　扩大“四上”企业数量。落实“小升规”激励和支持政策，推动小微企业提质升级。新增规模以上工业企业300户以上、限额以上批零住餐企业100户以上、规模以上服务企业100户以上、资质等级建筑业及房地产企业150户以上。培育更多总部在青岛的全国性企业和跨国公司，支持更多企业进入全省100强、中国500强和世界500强。

　　扩大上市公司数量。支持大中型企业实施规范化公司制改制，完成有限公司改制700家、股份公司改制200家以上。引导企业精准对接资本市场，鼓励企业在国内外证券交易所上市或在场外市场挂牌交易。

　　支持民营企业发展。落实各项支持政策，增强民营企业投资和发展信心。放宽市场准入，消除显性或隐性障碍，强化金融支撑，激发民营企业活力，促进民间投资稳定增长。落实保护产权政策，依法保护企业合法权益。完善中小企业服务体系，发挥工商联和各类商（协）会作用，畅通政府与民营企业沟通渠道，健全企业家参与涉企政策制定机制。深入构建亲清新型政商关系，尊重企业和企业家，尊重产业工人，尊重各类劳动者，激发和保护企业家精神。

　　（三）抓好创新驱动。充分发挥创新对建设现代化经济体系的战略支撑作用，努力以创新引领率先实现优化发展，建设我国沿海重要的创新中心。

　　发挥好创新主体作用。促进创新要素向企业集聚，加快培育具有强大创新能力的行业引领者。引导企业加大研发投入，支持企业建设国家重点实验室、工程研究中心、企业技术中心、工业设计中心、制造业创新中心等高端创新平台，承担国家重大科技项目。深入实施科技企业培育“百千万”工程。高新技术企业超过2500家。

　　发挥好政府作用。完善技术交易市场体系，积极推动形式多样的产学研对接洽谈。着眼企业需求，建立健全公共服务平台，支持发展专业服务机构，完善中小企业技术创新社会化服务体系。落实各项优惠政策，推动科技与金融紧密结合，发展科技金融专营机构，强化科技信贷支持。加大企业创新产品（服务）采购支持力度。

　　提高产学研协同创新水平。支持企业联合大学和研究机构组建产业技术创新联盟或设立研发机构，促进协同创新和大型科学仪器设备开放共享。鼓励驻青大学和研究机构深度融入我市发展，不断推出支持产业发展的专业或课程，加强支持产业发展的新技术、新产品开发应用。优化转移转化机制，促进国内外大学和研究机构更多科技成果在我市转化，打造国家科技成果转移转化示范区。

　　（四）抓好深化改革。把牢方向，强化担当，狠抓落实，以完善产权制度和要素市场化配置为重点全面深化改革，加快实现产权有效激励、要素自由流动、价格反应灵活、竞争公平有序、企业优胜劣汰。

　　深化农村改革。加快农村集体产权制度改革，力争80%以上村庄完成改革任务。建立集体资产保值增值长效机制。深化土地“三权”分置改革，保障农民土地收益。巩固和完善农村基本经营制度，完成土地承包经营权确权登记颁证任务。完善农业支持保护制度，鼓励发展多种形式规模经营。深化以绿色生态为导向的农业补贴制度改革。加强涉农资金统筹整合。

　　深化国企国资改革。改革和完善国有资产监管体制机制。推进经营性国有资产统一监管改革，强化对国有企业和国有资本审计监督。鼓励和支持国有企业并购重组，促进国有资本向重点行业、关键领域和优势企业集中，推动国有资本做强做优做大。推动投融资平台向完全市场化经营企业转变。加大混合所有制改革力度，支持国有企业多渠道增资扩股和进行规范化公司制改制。实施“一企一策”，培育具有国际竞争力的企业集团。

　　深化财税金融改革。强化重点税源培植，提高财政收入的税收占比，新增财力重点用于民生改善、现代化经济体系建设和美丽青岛建设。深化财政事权和支出责任划分改革。全面实施专项资金清理整合和绩效管理。支持规范使用PPP模式建设基础设施和市政项目。积极争取税收优惠政策，支持我市创业投资、天使投资快速发展。加强政府性债务管理，严控债务规模。有序有效推进金融创新，加强风险监测和信息共享，强化对各类地方法人金融机构监管，强化薄弱环节监管，坚决打击非法集资以及各种非法金融活动，牢牢守住不发生区域性金融风险的底线，坚决打好防范化解金融风险攻坚战。

　　（五）抓好全面开放。深入实施国际城市战略，推进企业国际化、市场国际化、产业国际化、园区国际化、城市国际化，构建开放型经济新体制，打造全方位深层次立体式开放新格局。

　　大力开展招商引资。加强督查考核，健全奖惩体系，促使各级各部门各方面以扎实行动积极实施招商引资。继续深入开展“千企招商大走访”和现代产业精准招商行动，开展好主题招商、委托招商、中介招商、以商招商和产业链招商，大力推进专业团队专题招商，有针对性地引进一批规模大、产业链拓展和协同性强、技术水平和财税贡献高的项目。促进内外资融合发展，实际利用外资突破80亿美元，到位内资超过2100亿元。

　　积极建设贸易强市。更加注重提高出口质量和附加值，鼓励先进技术、关键设备和零部件、优质消费品进口，促进货物贸易、服务贸易进出口稳定增长，创建区域性国际贸易中心。推进跨境电子商务综合试验区建设，搭建好综合服务平台，用好零售进口过渡期政策，建设好跨境电商产业重点园区。建立一批“海外仓”和境外展示中心。积极拓展境外投资贸易，推进服务贸易创新发展，打造服务外包城市品牌。培育共享出口、市场采购贸易等外贸新模式。

　　积极融入“一带一路”建设。发挥节点支点城市作用，坚持陆海统筹，突出经贸主线，建设“一带一路”综合枢纽城市。推进欧亚经贸合作产业园建设，打造更多面向重点国家的国际经贸产业合作平台，打通“一带一路”沿线经济贸易通道。加快境外经贸合作园区建设。支持企业通过对外投资增强研发能力、优化全球布局、打造国际品牌。高水平搭建东亚海洋合作平台，建成永久性会址。

　　积极扩大全方位开放。落实“国际化+”行动计划，提升全面开放水平。积极争取国家关于投资和贸易自由化便利化的改革安排，用好国家自贸区战略地方经贸合作推进工作机制，复制推广先进省市自贸试验区经验。加快各行业各领域对外开放，统筹一二三产业全产业链对外开放。对接国际标准，加快建设国际化社区、国际学校、国际（涉外）医院和城市综合服务体系。深化与国际友好城市、友好合作关系城市和国际经济合作伙伴城市的交流合作。加强与沿黄流域、沿海区域的交流合作，强化与省内各市和周边地区的融合发展。

　　今年，上海合作组织峰会将在我市举办，这是党的十九大之后我国举办的一次重大主场外交活动，是以习近平同志为核心的党中央对我们的信任，也给青岛向世界展示新形象、为发展增添新活力提供了难得的历史性机遇。我们一定高度重视、精心准备，扎实做好有关服务保障工作，为办成一次精彩圆满的盛会作出青岛贡献。

　　（六）抓好营商环境建设。着力在优化政务服务、维护公平竞争、促进投资贸易和工作生活便利化等方面下功夫，营造让企业和居民个人满意的一流环境。

　　深化“放管服”改革。加强政务信息系统整合共享，加快推行网上审批。实施建设工程项目“大容缺、大并联”等改革创新，实现全流程联审联办。放宽登记注册条件，实行“非禁即入”。推进证照分离，推行工商登记全程电子化改革。对企业开办、纳税、施工许可、水电气报装、不动产登记等事项大幅精简审批、压缩办理时间。推进“双随机一公开”监管，对新业态采取更加包容审慎的监管制度。

　　打造公平透明可预期的营商环境。全面实施市场准入负面清单制度和公平竞争审查制度。加快建立营商环境评价机制。深化“四减一降”，放宽市场、行业、从业准入。保护和规范竞争，完善部门协同机制，规范市场秩序。加快要素价格市场化改革，统筹协调水煤电油气运等生产要素，保障企业生产经营。全面推行警务、司法行政公开。推进诚信建设，建立失信守信黑红名单及公布制度，完善激励惩戒机制。

　　努力为居民个人办事提供最大便利。持续开展简证便民行动，清理各种证明和手续。大力提升与群众生活密切相关的公共服务质量和效率。推进“互联网+政务服务”，努力让“最多跑一次”和“零跑腿”成为常态。

　　四、致力于城乡环境品质提升，加快时尚美丽青岛建设

　　落实区域协调发展战略、乡村振兴战略和可持续发展战略，推进建设美丽青岛三年行动，增强城市对外辐射和资源集聚能力，使青岛成为宜居宜业宜游、让本地人自豪让外地人向往的时尚美丽的海滨城市。

　　（一）推动城市现代化建设上新水平。推进城市规划科学化、建设精品化、管理精细化、运营高效化，打造智慧城市、绿色城市、人文城市，提升城市品质和竞争力。

　　科学规划。遵循明智规划理念，有序推进“多规合一”，绘制市域空间“一张蓝图”。启动编制《青岛市城市总体规划（2021-2035年）》。完成中心城区总体城市设计。加强专项规划的编制和落实。加快乡村建设规划及村庄规划编制。推进市政交通规划编制，推动有助于解决交通拥堵、停车难等问题的市政设施建设。

　　建设精品。将精品意识贯穿到城市建设各领域，全面争创优质工程。加快胶东国际机场建设，主体工程具备验收条件。力争济青高铁和青连铁路建成通车、潍莱高铁全面开工。加快地铁8号线建设，开通地铁11号、13号线。加快新机场高速一期和济青高速改扩建工程建设，实现龙青高速莱西至龙口段通车。推进普通国省道养护大中修和安防工程，把农村公路建好管好护好运营好。新建改建绿地200公顷，改建口袋公园景观节点100处以上。加强地下空间普查和综合利用。新建海绵城市30平方公里。坚持不懈推进“厕所革命”，巩固农村改厕成果，市区新建改建公厕230座。

　　精细管理。全面推行街长制，推进多元参与、齐抓共管。加强市容环境整治，全面清理楼宇广告和各种违法户外广告，规范门头牌匾，提升保洁水平、绿化档次及晚间亮化品质。突出规范化、人性化，全面整治道路及附属设施。基本完成违法建设治理。强化道路交通秩序整治，建立智能化交通管理服务系统。开辟调整市区公交线路50条，推进候车亭提档升级。多渠道有效增加停车位，强化道路停车管理。加强共享单车综合管理。

　　高效运营。扩大城市运营领域政府购买服务范围，市场能做好的事情坚决交给市场。强化水资源优化配置和统一调度，积极准备、尽早启动黄水东调青岛承接工程，加大海水淡化能力建设，保障城市供水安全。推进水厂、输配水管网建设，提升饮用水品质。推进再生水利用工程。全面推行非居民用水差别化价格制度。开工建设胶州湾天然气海底管道，管道天然气乡镇驻地覆盖率达到90%。推进“世界一流城市配电网”建设。市区全面推行生活垃圾分类，推动农村垃圾分类试点，提升生活垃圾分类运输和处理能力。在建垃圾焚烧项目尽快投运，增量垃圾全部焚烧。做好迎接国家卫生城市复审工作。

　　建设全国领先的智慧城市。开展智慧城市评价标准应用工作。编制通信基础设施专项规划，加快信息网络基础设施升级。积极推进国际通信业务出入口局设立工作。优化整合政务大数据和云计算中心，推动技术融合、业务融合、数据融合，打通信息壁垒。加强信息安全建设。深入推进智慧化建设，提升面向企业和市民、游客的各种政务服务和市场服务的智慧化水平。打造便捷支付城市。

　　（二）推动区域协调发展上新水平。加强统筹协调，完善政策措施，着力增强区域发展的系统性，拓展发展新空间，提高全域发展水平。

　　推进重点功能区创新发展。落实山东省青岛西海岸新区条例，鼓励新区先行先试，加快转型升级。提升高新区发展水平，在山东半岛国家自主创新示范区建设中发挥更大作用。推进蓝谷省级区域创新中心建设，打造面向全球的海洋创新高地。支持保税港区全域封关和平行车进口试点落地。推进财富管理金融综合改革试验区建设，财富产品交易中心尽早运营，争取在人民币跨境投融资等方面有新突破。加快胶东临空经济示范区规划建设，打造区域性航空枢纽、高端临空产业基地。加快军地共享基础设施建设，培育共生型军民融合产业，打造国家军民融合创新示范区。推动出口加工区向综合保税区转型。加快开发区和各产业园区转型升级，打造改革开放新高地、创新发展新龙头。

　　激发区域发展新活力。深入推进老城区有机更新，优化城市空间、产业和功能布局，实现资源、要素、产业、项目合理配置。加快市南时尚美丽中心区、市北国际邮轮港城、李沧国际院士港及产业核心区、崂山虚拟现实产业基地等重点产业载体建设，支持城阳国际创新生态城、即墨汽车产业城建设发展。支持胶州空港综合保税区项目建设，深化平度国家中小城市综合改革试点，推进莱西新能源汽车基地建设，提升县域经济能级。加快试点小城市建设，培育一批特色小镇。深化户籍制度改革，放宽落户条件。统筹城乡公共服务，推进基本公共服务均等化。

　　（三）推动农业农村现代化建设上新水平。按照产业兴旺、生态宜居、乡风文明、治理有效、生活富裕的总要求，科学制定乡村振兴战略规划，建立健全城乡融合发展体制机制和政策体系，努力让农业成为有奔头的产业，让农民成为有吸引力的职业，让农村成为安居乐业的美丽家园。

　　加快推进农业现代化。坚持质量兴农、绿色兴农，深化农业供给侧结构性改革，加快构建现代农业产业体系、生产体系、经营体系。培育充满活力的新型经营主体，新增规范化农民合作社、家庭农场、农业社会化服务组织500家以上。促进农村一二三产业深度融合，创建农业“新六产”示范区。发展加工农业，新增销售收入过亿元企业10家。发展园区农业，打造一批现代农业产业园、农村创业创新园和标准化规范化畜禽养殖场。加快发展休闲旅游农业。推进“互联网+现代农业”，培育一批农产品电商示范镇。实施农业科技创新工程，壮大农业科技创新推广团队。加快种子产业发展。加强粮食流通工作。落实好“菜篮子”市长负责制。新建农民田间学校75所，培训新型职业农民1.5万人。

　　加快推进农村现代化。实施农村“七改”工程，推动农村基础设施提档升级。强化农村环境整治,改善农村人居环境。加强农业面源污染治理，实现化肥农药施用总量零增长。推动畜牧养殖规模化并向优势产区集中，配建畜禽养殖废弃物处理设施100处。推进美丽乡村标准化创建行动，新建一批农村新型示范社区、美丽乡村示范村和达标村。

　　加快推进乡村治理体系和治理能力现代化。加强农村社区服务中心建设，打造服务“三农”主阵地。以民主议事为前提，持续推进村级公益事业建设一事一议财政奖补。健全自治、法治、德治相结合的乡村治理体系，激发村民参与乡村事务积极性，建设乡村共同体。支持村集体经济组织发展物业经济、资源经济和服务经济。做好村（居）民委员会换届选举工作。

　　（四）推动生态文明建设上新水平。坚守绿水青山就是金山银山的理念，实施生态保护修复重大工程，打好污染防治攻坚战，努力提供更多优质生态产品。

　　强化环境污染治理。全面完成中央环保督察反馈问题整改。深入实施大气污染防治行动，加强工业污染、机动车船排放污染、城市扬尘等专项治理。实施水污染防治行动，加强黑臭水体和污水直排等问题治理。规划建设重点河流生态补水工程，建成启用张村河净水厂。加大大沽河保护管理力度。实施“蓝色海湾”治理工程，推进胶州湾、西海岸国家级海洋公园建设。严格落实河（湖）长制、湾长制，加大河库（湖）、海域综合整治和保护力度。推进土壤污染防治，加强关闭搬迁企业场地再利用环境管理。

　　强化资源高效利用。发展节能环保、清洁生产和清洁能源产业，争取列入国家节能量、用能权交易城市，积极开展碳市场交易。推行国家绿色制造标准体系，实施省级以上园区循环化改造，开展生产者责任延伸制度试点。扩大海水源、污水源、空气源热泵和工业余热等清洁能源利用，新增供热能力2000万平方米。加强民用散煤清洁化治理。发挥中德生态园被动房示范社区引领作用，发展装配式建筑和绿色建筑。推进绿色建设科技城试点。实施节能保暖改造500万平方米。依法处置闲置土地，盘活低效用地。开展节约型机关、绿色家庭、绿色学校、绿色社区和绿色出行等创建行动。

　　强化生态系统保护。落实主体功能区规划，推进主体功能区建设。加大生态红线保护力度，坚决守住生态功能保障基线、环境质量安全底线、自然资源利用上线。提升自然保护区规范化建设管理水平。推进“绿满青岛”国土绿化行动，新造林12万亩。加快青岛北部生态屏障区建设。加强湿地保护。强化永久基本农田特殊保护。

　　强化环保监管。落实规划环评与建设项目环评制度。开展环境安全隐患排查整治，加强重点区域环境风险综合管控。推进环保机构监测监察执法垂直管理制度改革。加强部门联合监管执法，严惩环境违法行为。健全环保信用评价制度。全面推行领导干部自然资源资产离任审计。

　　五、致力于更加富有活力，加快城市软实力建设

　　坚定实施科教兴市、人才强市和创新驱动发展战略，大力实施质量强市、标准化和品牌战略，全面提升城市软实力、增强核心竞争力。

　　（一）坚持全面创新，建设创新强市。激发全社会创新活力和创造潜能，建设创新资源富集、创新人才汇聚、创新环境一流、创新活力迸发的创新型城市。

　　推进“双创”行动。拓展市场化、专业化众创空间，支持企业、大学和研究机构等建设专业孵化器和“双创”平台，推广“人人创客”等“双创”新模式，开放共享创新资源，打造大中小企业协同“双创”新格局。引进一批国内外高水平、专业化孵化机构。实施大众创业工程，构建覆盖城乡的创业孵化体系。促进平台经济、众包经济、共享经济等新业态健康发展。积极推进商业模式创新，推动各领域管理创新、制度创新。

　　推动形成更多创新成果。加快国家、省、市认定的各类创新平台建设，打造高水平科技创新载体和产业创新基地。发挥好驻青大学和研究机构以及行业协会、学会的作用。调动各级政府和广大企业积极性，继续大力引进域外大学、研究机构在青设立分支机构。

　　围绕我市产业升级需要，积极参与承担国家重点研发计划和科技创新2030重大科技工程。加快重点领域核心技术攻关，形成一批具有自主知识产权的核心产品和产业支撑技术，打造一批专利成果。加强知识产权创造、保护和运用。

　　推进创新生态优化。加快建立以知识价值为导向的分配激励机制，激发广大科研人员的积极性、创造性。加强创新教育，倡导鼓励创新、尊重个性、宽容失败、开放包容的创新文化，营造有利于创新创业的良好氛围，让创新在全社会蔚然成风。建设知识型、技能型、创新型劳动者大军，弘扬劳模精神和工匠精神，营造劳动光荣的社会风尚和精益求精的敬业风气。

　　（二）坚持人才优先，建设人才强市。坚持人才“第一资源”，实施更加积极、开放、有效的人才政策，建立更具竞争力的人才发展制度,聚天下英才而用之，让青岛成为海内外优秀人才创新创业的热土。

　　优化人才成长环境。深化人才改革试验区试点，加快人才创业园等园区发展。利用好现有孵化器和众创空间，优化成果转化、资金服务等政策机制，促进各类人才脱颖而出。深化职称制度改革，开展技能人才与工程技术人才贯通发展试点。实施人才安居工程，推进规模化人才公寓供应。

　　加强人才引进培养。实施百万人才集聚行动，让更多人才进得来、留得住、干得好。突出产业需求，突出“高精尖缺”，实施顶尖人才、科技领军人才等人才工程，引进培养具有国际水平的战略科技人才、科技领军人才、青年科技人才和高水平创新团队。高度重视并积极支持实用型工程技术人才的培养引进。落实外籍雇员管理暂行办法。探索建设海外高层次人才离岸创新创业基地。

　　（三）坚定文化自信，建设文化强市。坚持文化事业和文化产业协调发展，推动优秀传统文化创造性转化、创新性发展，促进文化繁荣兴盛，增强文化软实力。

　　深化文明城市建设。强化教育引导、实践养成、制度保障，把社会主义核心价值观融入社会发展各方面。以灵活多样的形式弘扬城市的科学精神、人文精神和艺术精神。开展移风易俗、弘扬时代新风行动。深化中国特色社会主义和中国梦宣传教育，推进“四德工程”和未成年人思想道德建设。启动第六轮全国文明城市创建，深化群众性精神文明创建活动。落实青岛市文明行为促进条例，让“您懂得，别忘了”文明温馨提示深入人心。引导市民和游客从排队乘车、文明驾车、不乱穿马路、不乱扔垃圾等小事做起，自觉养成文明出行习惯。推进志愿服务制度化。繁荣发展哲学社会科学。支持特色新型高端智库发展。深化双拥共建，做好国防教育和民兵预备役工作。做好司法行政、民族宗教、侨务、对台工作，推动统计、档案、史志、仲裁事业发展，提高人防、气象、防震减灾工作水平。加强法律援助工作。推进老龄、残疾人、红十字、慈善事业不断进步，促进妇女儿童、青少年事业健康发展。

　　提升城市文化魅力。加强城市优秀传统文化的挖掘和阐发，彰显开放包容、海纳百川、创新求变的文化特点。加强具有青岛特色、与时俱进的海洋文化建设。充分挖掘具有悠久历史的传统地域文化资源，加强古遗址、传统村落、历史文化街区、工业遗产等文化遗产的保护利用。深入挖掘革命历史重要事件、重要人物和重要遗址，推出中共青岛支部旧址、大泽山抗日战争遗址、青即战役旧址等一批红色文化之旅，打造半岛红色文化传承创新区。加快近现代建筑活化再利用，推进名人故居保护修缮和有序开放。深化“东亚文化之都”和“一带一路”文化交流，提高青岛文化辐射力。实施文化惠民工程，加强公共文化服务设施建设，办好市民文化艺术节，做好国家文化消费试点，开展全民阅读活动。

　　增强城市文化实力。坚持思想精深、艺术精湛、制作精良相统一，打造具有全国影响力和国际美誉度的优秀文化产品。挖掘“电影之都”的产业效应，引进培育一批高端影视企业，加强国际电影及相关创意领域合作，筹办上海合作组织国家电影节等国际电影节会，打造有影响力的影视产业基地。大力发展文化创意产业，推进媒体深度融合，培育新型文化业态，形成一批有影响力的文创企业和园区。办好亚太地区工艺美术大会。

　　(四)坚持内外兼修，建设品牌强市。传承老品牌承载的工匠精神和文化基因，以标准提档、质量升级、品牌增效为着力点，提升品牌之都影响力。

　　夯实品牌建设基础。开展质量提升行动和对标达标专项行动，引导企业提高产品和服务质量。实施“标准化+”行动，鼓励企业、研究机构、行业组织等参与各类标准制定。实施商标战略，鼓励市场主体充分运用商标创品牌。促进“老字号”振兴发展，打造更多“百年老店”。

　　打造行业著名品牌。发挥好海尔、海信、青岛啤酒等著名品牌的模范带动作用，实施品牌升级行动，打造更多引领消费潮流、附加值高、竞争力强的产品（服务）品牌和商标。抓好工业企业品牌培育试点示范，争取在传统行业和新兴领域都新增一批知名品牌。加强服务业标准体系建设，努力在重点领域培育一批品牌企业和项目。推动工程建设领域标准化、集成化发展，培育一批高端品牌。新增“三品一标”农产品60个，创建国家农产品质量安全市。积极打造公共服务品牌。

　　加强城市综合营销。以扎实有效的措施和办法，抓好面向国内外的城市综合营销，讲好青岛故事，讲全青岛优势，讲足青岛能力，彰显青岛形象。加强政府与企业之间的协同，引导企业制定品牌战略规划，开展差异化品牌形象传播。加强与国内外知名媒体、研究和中介机构、行业协会的沟通交流，充分利用各类媒体和平台，加强对外宣传推介，提升城市影响力和吸引力。

　　六、致力于满足人民美好生活需要，加快民生和社会事业建设

　　坚持把人民对美好生活的向往作为始终不渝的奋斗目标，不断改善民生工作、发展社会事业，努力使群众有更多获得感、幸福感、安全感。

　　（一）努力使群众享有更优的教育。坚持教育优先，发展公平而有质量的教育，加快教育现代化进程。坚持立德树人，发展素质教育。实施学前教育行动计划，建设普惠性幼儿园60所。实施城乡义务教育一体化发展工程，建设中小学30所。建设中小学标准化食堂100所。深化现代学徒制改革试点和中高职一体化发展改革，培育一批高水平职业院校和优势专业。支持驻青大学加快发展。推动已签约大学驻青机构加快建设，尽快开展教学研究活动。办好特殊教育，建成盲校新校。支持和规范社会力量兴办教育，提供多样化、高水平教育服务。推进家庭教育普及工程。办好继续教育。加快教育信息化建设。加强师德师风建设，培养高素质教师队伍。

　　（二）努力使群众享有更高的收入。坚持就业优先战略和积极就业政策，鼓励以创业带动就业。实施高校毕业生就业创业促进计划和基层成长计划，做好退役军人安置工作，抓好进城务工人员、城镇就业困难人员等群体就业，实现城镇新增就业30万人，政策性扶持创业1.5万人。实施职业技能培训3万人。完善劳动关系协商协调机制。健全农民工工资支付督查和治理机制。通过支持农民就业创业、农业产业化、发展农村电商等综合性措施，保持农民增收势头。

　　（三）努力使群众享有更好的保障和福利。推广社保卡“一卡通”。提高居民医疗保险财政补助标准和保障待遇。健全长期护理保险保障制度。扩大跨省异地就医联网结算定点医疗机构范围。完善医疗救助制度。解决好城镇困难居民的生活保障。完善农村留守儿童和妇女、老人、残疾人关爱服务体系。提高孤儿生活保障标准。

　　坚持房子是用来住的、不是用来炒的定位，严厉打击扰乱房地产市场秩序的违法行为，注重长效机制建设，培育和发展住房租赁市场，积极开展租赁住房建设，探索共有产权住房制度，加快建立多主体供给、多渠道保障、租购并举的住房制度。实施住房保障7000套（户）。启动棚户区改造3.5万套（户）。完成老旧住宅小区综合整治211万平方米。改造农村危房1200户。

　　坚决打好脱贫攻坚战。瞄准特殊贫困人口精准帮扶，巩固扶贫成果。鼓励引导社会力量参与扶贫，抓好产业扶贫，深入开展金融、教育、健康等专项扶贫行动，实现10个市定贫困薄弱镇摘帽，完成全市农村脱贫攻坚任务，健全稳定脱贫长效机制。根据国家和省统一安排，继续做好对口支援和扶贫协作工作。

　　（四）努力使群众享有更高水平的医疗。实施健康青岛2030行动，为群众提供全方位全周期健康服务。深化公立医院改革，完善现代医院管理制度。规范医联体建设，推进分级诊疗，加强基层医疗机构标准化建设。加快10个重点医疗项目建设，积极引进高水平医院。实施“互联网+健康医疗”行动，促进群众就医便利化。新建18个院前急救站。做好国家中医药综合改革试验区和全国医养结合服务试点市工作。积极应对人口老龄化，建立养老综合服务互动平台，鼓励社会资本进入养老产业。开展妇幼健康、孕前优生等12项免费服务，解决好婴幼儿照护和儿童早期教育服务问题。健全社会心理服务体系，加强重症精神病患者集中收治。深入开展爱国卫生运动。建设100处以上居民身边的健身场地，广泛开展全民健身活动。承办好第24届省运会、第10届省残运会。

　　（五）努力使群众享有更广泛的参与。提高社会治理社会化、法治化、智能化、专业化水平，打造共建共治共享的社会治理格局。坚持开展“向市民报告、听市民意见、请市民评议”等系列活动，畅通政民互动渠道，保障公众对政府工作的知情权和参与权，支持市民关心城市发展、参与城市治理。完善社区治理体系，发挥社会组织作用，加强人民调解组织和机制建设。深化城乡社区警务建设，促进社区工作与警务工作高度融合。

　　（六）努力使群众享有更平安的环境。坚守安全生产、防火防汛、环境保护、食药安全、社会稳定等工作底线，推进平安青岛建设。强化安全发展理念，细化安全生产责任制，深化安全生产改革创新，抓好各领域专项整治，坚决遏制重特大安全事故。加快化工产业安全生产转型升级，深化危险化学品综合治理。加强防灾减灾救灾能力建设，健全应急管理体系，开展中小学生应急安全体验活动，培训应急“第一响应人”2万人。推进“食安青岛”建设，开展食品安全定性定量检测6.5万批次。做好信访稳定工作。加强社会治安综合治理，推进立体化、智能化防控体系建设，严厉打击各类违法犯罪，加强反恐禁毒工作，完善网络安全管控体系，保障群众人身权、财产权、人格权，保持良好的社会秩序。

　　七、致力于打造人民满意的服务型政府，加强政府工作人员本领和作风建设

　　深入学习贯彻党的十九大精神，全面增强学习本领、政治领导本领、改革创新本领、科学发展本领、依法执政本领、群众工作本领、狠抓落实本领、驾驭风险本领，增强政府的公信力和执行力。

　　（一）提高政治站位。把政治建设放在政府自身建设的首位，把党的领导贯穿政府工作全过程各方面。牢固树立“四个意识”，坚定“四个自信”，坚决维护习近平总书记的核心地位和领袖权威，坚决维护以习近平同志为核心的党中央权威和集中统一领导。认真开展“不忘初心、牢记使命”主题教育，深入推进“两学一做”学习教育常态化制度化。

　　（二）提高法治政府建设水平。依法全面履行政府职能。严格执行市人大及其常委会决议决定，落实重大事项向人大及其常委会报告、向政协通报制度，自觉接受市人大的法律监督和工作监督，主动接受市政协民主监督。做好建议提案办理工作。认真听取各民主党派、工商联、无党派人士和人民团体意见。推进科学民主依法决策。完善政府法律顾问工作机制。提高政府立法工作质量。健全行政执法体制机制，严格规范公正文明执法。完善行政争议多元化解机制。推进公共法律服务平台规范化建设。落实普法责任制。着力提高政府工作人员法治思维和依法行政能力。全面推进政务公开、信息公开，强化对行政权力的制约监督。

　　（三）提高抓落实水平。天下难事必作于易，天下大事必作于细。各级各部门和政府工作人员一定要崇尚实干、力戒空谈、精准发力，增强落细落小落地落实能力，以真抓的实劲、敢抓的狠劲、善抓的巧劲、长抓的韧劲，让改革发展稳定各项任务落下去，让惠及百姓的各项工作实起来。持续开展市政府领导班子成员联系代表委员、联系企业、联系重点项目和走访企业活动。拜群众为师，向群众学习，深入一线调查研究，真正把功夫下到察实情、出实招、办实事、求实效上。注重培养政府工作人员的专业能力、专业精神，不断提高服务意识和服务水平。关心关爱基层工作人员，主动为他们排忧解难。

　　（四）提高从严治政水平。严格落实主体责任，扎实推进政府系统全面从严治党。锲而不舍抓好作风建设，巩固拓展落实中央八项规定精神成果，持之以恒纠“四风”，坚决反对形式主义、官僚主义。深化标本兼治，巩固发展反腐败斗争压倒性态势。加大对侵害群众利益的不正之风和腐败问题整治力度。严肃财经纪律和各项工作纪律，强化财政资金和公共资源使用的监督管理。加大审计监督和审计查处问题的整改力度。整肃庸政懒政怠政和乱作为，让失责必问、问责必严成为常态。完善激励机制和容错纠错机制，旗帜鲜明为那些敢于担当、踏实做事、不谋私利的干部撑腰鼓劲。

　　各位代表！使命呼唤担当，实干赢得未来。让我们更加紧密地团结在以习近平同志为核心的党中央周围，以习近平新时代中国特色社会主义思想为指导，在中共青岛市委的坚强领导下，不忘初心、牢记使命，锐意进取、埋头苦干，为实现在社会主义现代化新征程中率先走在前列努力奋斗！
